# El futuro de la medicina rural

**DOI:** 10.1016/j.aprim.2024.102973

**Published:** 2024-05-29

**Authors:** Sonia Jover Garrido, Carme Planes

**Affiliations:** a Facultad de Medicina y Ciencias de la Salud, Universitat de Barcelona, Hospital Clínic, Barcelona, España; b Servicio de Medicina Interna, Hospital Universitari Sagrat Cor. Quirón Salud, Barcelona, España

*Sr. Editor,*

En el seno de las comunidades rurales nace una necesidad racional y a menudo descuidada de una atención médica suficiente. La disparidad en la atención médica entre las áreas rurales y urbanas produce escasez de recursos y socava la salud general de la comunidad rural.

Los beneficios profesionales y personales que ofrece la práctica de la medicina rural son sobradamente representados por aquellos que la han ejercido a lo largo de su vida laboral^1^.

Sin embargo, las dudas sembradas con respecto a la Atención Primaria durante los últimos años dificultan que los estudiantes de medicina tengan en cuenta opciones profesionales que permitan aplicar en el ámbito rural los conocimientos adquiridos.

Las estrategias para promover la medicina rural van a ser, si no lo son ya, una necesidad emergente en el ámbito académico para revertir la tendencia actual. En España, el 28% de los médicos rurales se jubilará en los próximos cinco años, dejando 4.500 plazas libres para los estudiantes que se están formando. Identificar aquellos factores que se asocian a una mayor tendencia a escoger la medicina rural por parte de los jóvenes es una estrategia útil para nuestro futuro.

En estudios realizados en estudiantes sobre la intención de ejercer la medicina rural no se han encontrado diferencias significativas entre sexos, etnias o edades. Sin embargo, la intención de ejercer esta práctica clínica es significativamente mayor en estudiantes criados en el ámbito rural o con parejas y familiares cercanos que se hayan desarrollado en el mismo^2^.

Los problemas sustanciales que confrontan a los estudiantes con la medicina rural están bien representados por la bibliografía. La escasez de atractivo por la medicina rural se refleja en países como Estados Unidos, donde solo el 9% de los alumnos escoge la práctica rural a pesar de que el 20% vive en un entorno campestre.

Teniendo en cuenta que son muchos los elementos que pueden determinar la pérdida de interés por la práctica rural, algunas entidades ya han empezado a reforzar las prácticas clínicas fuera del área urbana entre sus estudiantes, obteniendo resultados prometedores. La experiencia en Atención Primaria rural durante la carrera desplaza la posibilidad de seguir esta trayectoria a mucho más probable en un 41% de los estudiantes^3^.

Los motivos que provocan una mayor aceptación de la medicina rural para el alumno incluyen la adquisición de una experiencia completa y mejores habilidades clínicas debido a la exposición constante con el paciente. Además, la mayoría de los estudiantes reportan beneficios de su rotación con médicos que trabajan en el entorno rural. La exposición prematura a la práctica clínica fuera del ámbito urbano promueve que los alumnos opten por la medicina rural.

En conclusión, es de vital importancia implementar nuevas estrategias para que los estudiantes de medicina opten por la medicina rural. Estas estrategias no solo ofrecerán oportunidades profesionales prometedoras, sino que también representarán el compromiso con la equidad en la salud y el desarrollo sostenible de nuestras zonas rurales, creando así un sistema de atención médica más justo y resiliente para todos.
